# Interleukins (Cytokines) as Biomarkers in Colorectal Cancer: Progression, Detection, and Monitoring

**DOI:** 10.3390/jcm12093127

**Published:** 2023-04-25

**Authors:** Sajida Maryam, Katarzyna Krukiewicz, Ihtisham Ul Haq, Awal Ayaz Khan, Galal Yahya, Simona Cavalu

**Affiliations:** 1 Department of Biosciences, COMSATS University Islamabad (CUI), Islamabad 44000, Pakistan; ihtishamulhaq535@gmail.com (I.U.H.); ayaz11.kust@gmail.com (A.A.K.); 2 Department of Physical Chemistry and Technology of Polymers, Silesian University of Technology, M. Strzody 9, 44-100 Gliwice, Poland; katarzyna.krukiewicz@polsl.pl; 3 Centre for Organic and Nanohybrid Electronics, Silesian University of Technology, Konarskiego 22B, 44-100 Gliwice, Poland; 4 Joint Doctoral School, Silesian University of Technology, Akademicka 2A, 44-100 Gliwice, Poland; 5 Department of Microbiology and Immunology, Faculty of Pharmacy, Zagazig University, Zagazig 44519, Al Sharqia, Egypt; galalyehia@zu.edu.eg; 6 Department of Molecular Genetics, Faculty of Biology, Technical University of Kaiserslautern, Paul-Ehrlich Str. 24, 67663 Kaiserslautern, Germany; 7 Faculty of Medicine and Pharmacy, University of Oradea, P-ta 1 Decembrie 10, 410087 Oradea, Romania; simona.cavalu@gmail.com

**Keywords:** colorectal cancer, immunity, biomarker, interleukins, cytokines, therapy, diagnostics

## Abstract

Cancer is the primary cause of death in economically developed countries and the second leading cause in developing countries. Colorectal cancer (CRC) is the third most common cause of cancer-related deaths worldwide. Risk factors for CRC include obesity, a diet low in fruits and vegetables, physical inactivity, and smoking. CRC has a poor prognosis, and there is a critical need for new diagnostic and prognostic biomarkers to reduce related deaths. Recently, studies have focused more on molecular testing to guide targeted treatments for CRC patients. The most crucial feature of activated immune cells is the production and release of growth factors and cytokines that modulate the inflammatory conditions in tumor tissues. The cytokine network is valuable for the prognosis and pathogenesis of colorectal cancer as they can aid in the cost-effective and non-invasive detection of cancer. A large number of interleukins (IL) released by the immune system at various stages of CRC can act as “biomarkers”. They play diverse functions in colorectal cancer, and include IL-4, IL-6, IL-8, IL-11, IL-17A, IL-22, IL-23, IL-33, TNF, TGF-β, and vascular endothelial growth factor (VEGF), which are pro-tumorigenic genes. However, there are an inadequate number of studies in this area considering its correlation with cytokine profiles that are clinically useful in diagnosing cancer. A better understanding of cytokine levels to establish diagnostic pathways entails an understanding of cytokine interactions and the regulation of their various biochemical signaling pathways in healthy individuals. This review provides a comprehensive summary of some interleukins as immunological biomarkers of CRC.

## 1. Introduction

Cancer is a condition where cells proliferate uncontrollably [1,2]. It is the leading cause of death in economically developed countries and the second leading cause of death in developing countries [3]. Colorectal cancer (CRC) is the third most common cause of cancer death globally, with an estimated 2.2 million new cases and 1.1 million deaths expected over the next decade [4]. CRC accounts for 9.2% of cancer-related deaths, making it the second leading cause of cancer death [5,6,7]. Both genetic and environmental factors play a role in CRC causation [8]. Chronic colitis due to inflammatory bowel disease (IBD) is also associated with an increased risk of CRC [9]. CRC has a poor prognosis, and there is a critical need for new diagnostic and prognostic biomarkers to reduce CRC-related deaths [10,11]. Cytokine networks are largely involved in the prognosis and pathogenesis of CRC [12]. Tumors express cytokines with both antitumor and pro-tumor properties [13]. Cytokines’ ability to stimulate oncogenic signaling has shifted research focus towards their role in promoting cell proliferation and survival during tumorigenesis [14]. Immune cells activated in response to the tumor produce and release growth factors and cytokines to regulate the inflammatory environment in tumor tissues [15]. These inflammatory cytokines direct DNA damage in the epithelium [16]. They imply the pathogenesis and prognosis of CRC [12]. Interleukins have distinct roles in CRC progression through tumorigenesis [14] and are also involved in tumor growth, cancer cell invasion, and metastasis, while also inhibiting cancer through complex pathways [15]. In recent years, interleukins have gained significant attention due to their distinct roles in providing a new and promising strategy for CRC treatment [17].

Interestingly, interleukins are not the only biomarkers for CRC. Since the gut microbiome has been shown to play a crucial role in the development of CRC, several studies have investigated the use of gut microbes as biomarkers for CRC [18,19,20]. The presence of *Fusobacterium nucleatum* and *Bacteroides fragilis* in tumors has been reported in 43% and 24% of patients, respectively. The detection of these bacterial species has been found to correlate with the overall bacterial load, but further analysis of microbial signatures via diversity profiling suggests that their detection may be indicative of a specific microbial profile [21]. Consequently, the presence of *F. nucleatum* and clbA+ bacteria in stool samples can be used as a predictive marker for CRC with a relatively high specificity and moderate sensitivity [22]. It is important to note that these non-invasive screening approaches are still under development and more research is needed to validate their results in larger patient populations and confirm their efficacy compared to current screening methods. Several studies have also investigated the use of miRNA as biomarkers for CRC. For example, a study found that a panel of miRNAs (e.g., miR-21, miR-31, miR-146a, and miR-192) had a sensitivity and a specificity for detecting CRC [23]. Even though these studies demonstrate the potential of miRNA as a non-invasive biomarker for CRC, more research is still needed to validate these findings in larger patient populations [24,25,26]. The type of cancer and stage of the disease can impact the miRNA signature. Furthermore, the methods used to detect miRNA, such as RT-qPCR and microarray analysis, can also contribute to inconsistencies in results [24,27].

The point-of-care (POC) detection of cancer biomarkers provides an effective means for early-stage diagnosis. The cytokines, especially IL-6 and IL-8, have been used as potential biomarkers allowing the diagnosis of various benign oral lesions from malignant ones. Various electrochemical and optical biosensors have been developed to detect interleukins [28]. Studies on clinical units and mouse models helped to reveal many cytokines that have a high correlation with specific diseases, overlaying an efficient way to develop Biology Collector (BIOCO) in clinics to promote the research in cytokine development platforms [29]. Cytokines can play a key role in disease management and diagnosis as an immune mediator, similarly to pathophysiology-based sepsis, where pro- and anti-inflammatory cytokines have a double-edged function [30]. Recent studies have focused on using molecular testing to guide targeted treatments for CRC patients, but introducing novel molecular tests into routine clinical practices remains a huge challenge [31]. Interleukins, which can be detected in blood circulation even in the case of localized tumors, have the potential to serve as biomarkers for detecting cancers, predicting disease outcomes, and managing therapeutic choices [32]. The detection of specific cytokines in blood can cost-effectively and non-invasively assist in the detection of cancer, as well as in determining the appropriate therapy and monitoring the progression of the disease [31,32,33,34]. This review aims to assess the role of interleukins as biomarkers for the early possible detection of colorectal cancer by searching advanced published literature and studies regarding immunological biomarkers’ expressions and roles in colorectal cancer using keywords; colorectal cancer, gastric cancer, cytokine network, diagnostics, and monitoring.

## 2. Molecular Pathways and Cytokine Role in CRC

The tumor microenvironment (TME) plays an important role in the initiation and growth of tumors into metastatic states [35]. Tumor cells secrete cytokines that stimulate self-proliferation, drug resistance, and activation and differentiation of other cells in the tumor microenvironment [36,37]. Neutrophils are triggered by complement immunity and release IL-1β, which can activate myeloid cells to produce IL-6 and increase the IL-17A response. IL-33 can be secreted by vascular endothelial cells and tumor cells [17]. The host’s immune response causes chronic inflammation from time to time, which leads to tumor growth primarily due to interactions between tumors, immune, and other cells moderated by cytokine [38,39]. Cytokines alert immune cells in the presence of tissue damage and infections to stimulate the cells to release more cytokine signals leading to chronic inflammation [38]. Chronic inflammation promotes diverse cytokine secretion during various stages and types of cancer [32]. It causes DNA damage and DNA mutation by reactive oxygen and nitrogen species, along with the alteration of the epigenome of the cells [40,41].

Immunological biomarkers can be predictive, prognostic, or both [42]. Biomarkers are genes, gene products, cells, enzymes, molecules, or hormones that can be detected in tissues and body fluids (blood, urine, etc.) [43]. Cancer biomarkers are usually produced by the tumor or other body cells in response to the tumor [44]. They can be used for early cancer detection and patient state prediction [45]. Certain biomarkers can reveal genetic changes in cancerous cells, such as gene rearrangements or amplifications, mutations, and cell division, enabling cancer detection and response prediction to various treatments [46,47]. Prognostic immune cells include tumor-infiltrating lymphocytes, eosinophils, neutrophilic granulocytes, macrophages, and dendritic cells [48,49,50,51]. The epithelium of a tumor activates stromal cells to release cytokines, such as transforming growth factor, and cell signaling factors to develop a microenvironment for tumor progression and metastasis [52,53,54].

**Figure 1:**
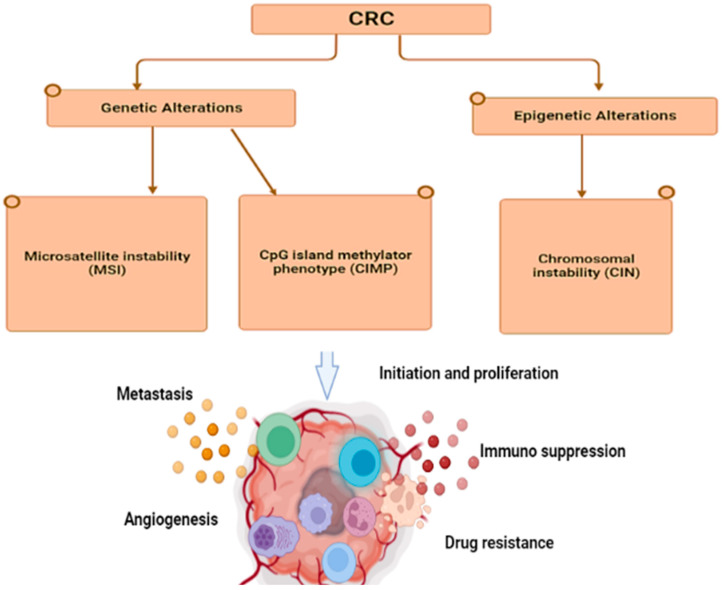
Molecular alterations and Genetic changes cause various pathways: chromosomal instability (CIN), microsatellite instability (MSI), and CpG island methylator phenotype (CIMP,) which lead to metastasis, angiogenesis, drug resistance, immunosuppression, and inflammation in tumors.

The development of colorectal cancer is also caused by specific mutations in oncogenes, tumor-suppressor genes, and genes associated with DNA repair mechanisms. However, approximately 70% of colorectal cancer cases follow a specific mutation and are transformed into a specific morphological sequence that starts with polyps and abnormal crypts, which then develop into early adenomas. The adenoma then progresses to advanced adenomas, eventually leading to colorectal cancer [55]. Therefore, genome instability is a fundamental feature of CRC. The pathogenic mechanisms leading to this situation can be divided into three different pathways (Figure 1): chromosomal instability (CIN), microsatellite instability (MSI), and CpG island methylator phenotype (CIMP) [56].

Mesenchymal cells of the gut, including endothelial cells, smooth muscle cells, mucosal immune cells, and subepithelial myofibroblasts, are combined with the tumor epithelial cells to regulate TME for the progression of CRC [57,58]. Table 1 represents main cancer cell types with the signaling pathways and target cells.

Many polymorphisms and somatically altered genes in CRC affect the KRAS- (kirsten rat sarcoma viral oncogene homolog), MYC- (cellular myelocytomatosis oncogene), Wnt-, mitogen-activated protein kinase (MAPK)-, or TGF-β/bone morphogenetic protein (BMP)-signaling pathways. For example, the nuclear factor κB (NF-κB), JNK, AP-1, and p38 MAPK pathways are activated by IL-1 receptor cytokine in CRC along with the activation of the Wnt signaling pathway by phosphorylation of GSK3β [12]. 

## 3. Advancements in Cytokine Detection and Monitoring Clinically

The study of cytokine secretion can lead to improved understanding of disease mechanisms and the development of new treatments. Elevated cytokine levels are a hallmark of many diseases such as diabetes, sepsis, cardiovascular diseases, neurodegenerative diseases, and cancers, and can be used as potential biomarkers [64,65,66,67,68]. A study found that a combination of IFN-γ, IP-10, ferritin, and 25-hydroxyvitamin D can be used to diagnose pediatric tuberculosis and to differentiate between TB and latent TB infection [69]. Inflammatory cytokines such as IL-6, IL-10, IL-1, TNF-α, and TGF-β are linked to the progression of heart failure and chronic kidney dysfunction, which are major global health problems with significant economic impacts [70]. Furthermore, inflammatory bowel diseases such as ulcerative colitis and Crohn’s disease result from an imbalance in pro- and anti-inflammatory cytokine interactions [29]. However, excessive cytokine release into the bloodstream from immune cells can cause cytokine release syndrome during immunotherapy and infections [71,72]. This makes detecting cytokines challenging due to their low concentration, instability, and complex networks [73]. Cytokine imbalances can also cause illnesses and prompt the need for precise and early treatment in severe conditions, so there is a demand for accurate, fast, and sensitive cytokine screening methods [74,75].

Common methods for cytokine quantification in clinical practice include ELISA and ELISpot [76]. Due to their crucial role in disease and human health, researchers are striving to develop tools for sensitive, multiplex cytokine detection [77]. For instance, recent studies have explored deployable devices with immunosensors on fiber optics [78] and stainless steel [79] for cytokine monitoring in mouse brain and spinal cords at pg/mL levels. An impedance aptasensor was developed for a highly sensitive detection of IL-6, with a detection limit of 1.6 pg/mL and a linear response of 5 pg/mL to 100 ng/mL [80]. A microfluidic technology based on single plex was designed to eliminate cross-reactivity and detect chemokines and cytokines in human and mouse samples [81]. Another chemiluminescent nanozyme immunoassay was developed for simultaneous detection of two chicken cytokines (IL-4 and IFN-γ) in serum samples with a range of 0.01–60 ng/mL for IFN-γ and IL-4, respectively, and detection limits of 2.9 pg/mL for IFN-γ and 3.2 pg/mL for IL-4 [82]. Clinical detection of serum biomarkers such as CA 19–9 and CA724 has a low specificity and sensitivity in gastric cancer diagnosis [83]. Studies show that the sensitivity, accuracy, and specificity of interleukins are as good as that of other markers such as carcinoembryonic antigen (CEA) and CA 19–9 [84]. This suggests that IL-6 may be a reliable biomarker for gastric cancer detection and diagnosis.

IL detection is commonly used as a biomarker for infections in clinical applications [85,86]. The most widely used tumor marker is a combination of ILs and CEA [87]. Tumor markers, found in body fluids, are widely used for early diagnosis, prognostic evaluation, and treatment monitoring of tumors [88,89], but CEA lacks specificity [90]. Combining inflammatory factors and tumor markers has shown a high potential in diagnosing pancreatic and CRC [91,92]. IL detection has also been analyzed for its individual role in the diagnosis of lung cancer and predicting lymph node metastasis [93]. They have shown promising results as a cancer biomarker in various types of cancer including gastric cancer, pancreatic cancer, and CRC. The use of ILs combined with other tumor markers such as CEA has the potential to enhance the accuracy of cancer diagnosis. In the case of thyroid cancer, the combination of IL-13 and IL-8 has been found to be effective in identifying the disease [94]. However, further research is needed to fully understand the genetic basis of interleukin levels as a cancer risk factor and to account for other factors that can impact the levels of interleukins in the body [95].

**Figure 2:**
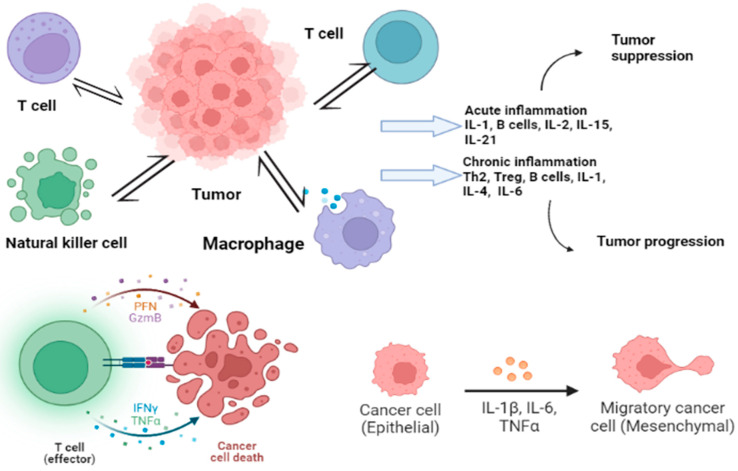
Immune cells influencing the tumor, leading to acute and chronic inflammation. Many cytokines (shown in form of colored dots in the figure) play their roles in this regard to either suppress or progress the tumor.

Alternation in the expression of interleukins such as IL-17, IL-22, and IL-1ß has been associated with various types of cancer [96,97]. Interleukins regulate the tumor microenvironment and are involved in tumor development and progression [98,99]. Not only locally, but their actions also occur distantly through circulation [100]. Interleukins promote cancer development by counteracting the immune response, mobilizing stromal, and immunosuppressive cells that support the tumor, inducing angiogenesis, and altering the response to therapeutic agents [101,102]. Their production is also triggered due to changes induced by cancer, alterations in metabolism, cell death, oxygen deficiency, and usage of anticancer drugs [102]. Figure 2 shows the immune response of cancer with cytokine release. The control of widespread cancer includes effective tools for the betterment of cancer burden by knowledge, early detection, suitable therapy along with a regular follow-up, and forecast measures by using cancer biomarkers [103]. The analysis of cytokines along with cancer-specific biomarkers has been put forward to improve cancer detection [32].

## 4. Cytokines’ Role in CRC

Some cytokines (other than interleukins) are also associated with the immune regulation of tumor cells and are highly expressed as compared to normal cells. They include forkhead box P3 (FOX P3), tumor necrosis factor-α (TNF-α), and interferon-gamma (IFN-γ).

### 4.1. Forkhead Box P3 (FOXP3)

The FoxP3 gene is expressed in regulatory T cells and is associated with cell development, transcription regulation, and DNA repair [104,105,106]. In addition to its basic role in immune responses, FOXP3 also plays a significant role in cancer development [107]. A high level of FOXP3 expression was observed in tumor cells compared to tumor-surrounding tissues, as detected through an immunohistochemistry assay [108]. FOXP3 levels are also higher in colorectal cancer tissues than in normal colorectal tissues [109], and its expression is associated with a poor prognosis compared to patients with low FOXP3 expression [110]. However, a high level of FOXP3 in tumor cells is associated with longer and disease-free survival [108]. Intra-tumoral CD4+ and FOXP3+ cell infiltration can be the most meaningful predictive factor in CRC patients [111]. Studies show that FOXP3 expression by cancer cells results in the secretion of cytokines such as IL-10 and TGFβ into the tumor microenvironment, which suppresses immunity [112].

### 4.2. Tumor Necrosis Factor-α (TNF-α)

Tumor necrosis factor-α (TNF-α) is a cytokine produced by macrophages and involved in various immune regulations [113]. It promotes the process of epithelial-to-mesenchymal transition (EMT) in colorectal cancer, thus promoting the metastasis of colorectal cancer [114,115,116]. Single-nucleotide polymorphisms in TNF-α genes are reportedly associated with the prognosis, therapy response, and survival of cancer patients [117]. High TNF-α gene expression is associated with Stage III and IV neoplasms compared to earlier tumor stages, and TNF-α expression is increased in the serum of CRC patients [118,119]. Statistical analysis shows that TNF-α mRNA expression levels are significantly higher in CRC compared to normal CRC tissue, and CRC patients with low TNF-α serum levels have a significantly higher survival rate compared to patients with high levels of TNF-α [118,120]. The examination of TNF-α levels in plasma can be used as a diagnostic factor for CRC instead of using other invasive tests [121].

### 4.3. Interferon-Gamma (IFN-γ) 

The genetic variations in interferon-gamma (IFN-γ) and its receptor (IFN-γR) subunits are strongly associated with the risk of colorectal cancer and patient survival after diagnosis [122]. IFN-γ is a major activator of macrophages and an inducer of Class II major histocompatibility complex (MHC) molecules [123], with immune regulatory, antiviral, and antitumor properties [124,125]. Studies have shown that the deficiency of IFN-γ or its receptor promotes the development of colorectal cancer, whereas its specific expression activates innate immunity and inhibits tumorigenesis [122,124]. The specific expression of IFN-γ activates innate immunity and inhibits tumorigenesis [124] However, IFN-γ signaling can also compromise antitumor immunity by inducing immune checkpoint inhibitory molecules on T and tumor cells [125]. Furthermore, IFN-γ acts as a cytotoxic cytokine and initiates apoptosis in tumor cells [126]. The *IFN-γ*/*Janus Kinase 2* (JAK)/*signal transducer and activators of transcription* (STAT) signaling pathway has been shown to induce programmed death-ligand 1 (PD-L1) expression in myeloid leukemia cells, pancreatic, and gastric cancer [127,128]. Increased PD-L1 expression levels have been found to be associated with a poor prognosis in patients with CRC [129]. Previous studies have also reported that PD-L1 expression on tumor-infiltrating immune cells is correlated with the survival of patients with CRC [130].

## 5. Interleukins in Colorectal Cancer

**Figure 3:**
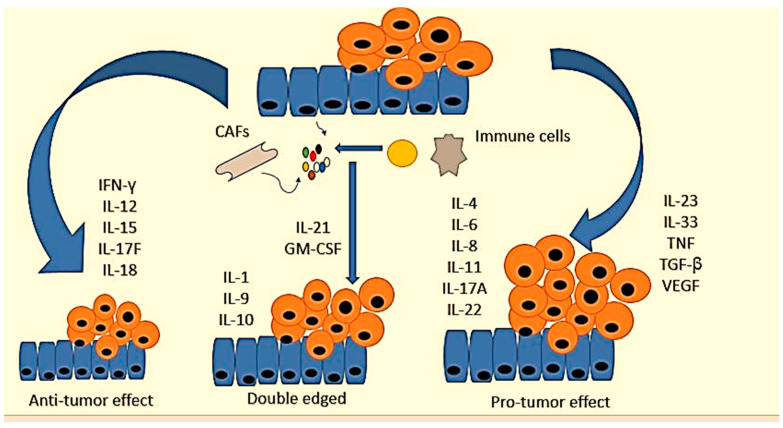
Cytokine networks in the pathogenesis of colorectal cancer. Cytokines expressed by tumor and/or stromal cells cluster to form networks with antitumor, pro-tumor, or bivalent properties. IFN-γ, interleukin-12 (IL-12), IL-15, IL-17F, and IL-18 inhibit CRC development. IL-4, IL-6, IL-8, IL-11, IL-17A, IL-22, IL-23, IL-33, TNF, TGF-β, and VEGF are pro-tumorigenic. The contribution of IL-1, IL-9 IL-10, IL-21, and GM-CSF to intestinal cancer remains unclear.

There are various interleukin families involved in CRC progression that have been studied as biomarkers (Table 2). They play different roles in colorectal cancer. For example, IFN-γ, interleukin-12 (IL-12), IL-15, IL-17F, and IL-18 inhibit CRC development [131,132,133,134]. On the other hand, IL-4, IL-6, IL-8, IL-11, IL-17A, IL-22, IL-23, IL-33, TNF, TGF-β, and *vascular endothelial growth factor* (VEGF) are pro-tumorigenic genes [135,136]. The contribution of IL-1, IL-9, IL-10, IL-21, and *granulocyte-macrophage colony-stimulating factor and sargramostim* (GM-CSF) to intestinal cancer remains unclear [135]. Figure 3 shows certain cytokines and their role in CRC.

### 5.1. Interleukin-1β

IL-1β is activated by immune response receptors to induce inflammatory responses [177]. As a pro-inflammatory cytokine, IL-1β is secreted by macrophages to promote cell proliferation [178,179]. Together with TNF-α, IL-1β is considered an “alarm cytokine” that triggers inflammatory responses by inducing other pro-inflammatory genes [180]. Polymorphisms in the IL-1β gene increase the risk of colon cancer development [181], while single-nucleotide polymorphisms (SNPs) associated with a high expression of IL-1Ra lead to better survival in patients with advanced CRC [182]. Mutations in the *Nucleotide Binding Oligomerization Domain* (NOD2) are also linked to severe forms of *Cluster of Differentiation* (CD) that secrete IL-1β, indicating its potential role in CRC progression [183]. Studies suggest that IL-1β promotes colon tumor growth by activating cancer stem cell (CSC) self-renewal and epithelial-to-mesenchymal transition (EMT) through the transcription factor *Zinc Finger E-box binding homeobox 1* (Zeb1) [184]. Therefore, IL-1β and Zeb1 could be potential therapeutic targets for colon cancer treatment. In one study, IL-1β was found to be significantly increased in CRC tissues compared to normal tissues, leading to the hypothesis that IL-1β plays a tumorigenic role in CRC and is associated with a higher rate of survival [185].

### 5.2. Interleukin-17

The identification of only six members of the IL-17 family has been achieved, i.e., IL-17A, IL-17B, IL-17C, IL-17D, IL-17E, and IL-17F [186]. However, the extensive study of only IL-17A and IL-17F has been performed related to CRC development [187]. IL-17 is being considered a promoter in the progression of colorectal cancer [188]. Studies show that the serum levels of IL-17A were elevated in CRC patients in comparison to healthy individuals [189] or in the circulating tumor cells which also predicted poor survival [190]. Furthermore, its gene expression is reported to be higher in tumor tissues compared to normal mucosa [171]. IL-17A expression heightens the adenoma-to-carcinoma sequence (mutational activation of cancer genes) in the intestinal epithelium of CRC patients [191]. Poor prognosis has been shown when there is a high expression of these genes associated with Th17 in CRC tissues [171]. In vitro, IL-17 and TNF-α synergistically promote carcinogenesis by stimulating glycolysis and growth factor production by CRC cells [192]. The study showed that CD4+ T-cell-derived IL-17 promotes tumorigenesis in the intestine of mice [193]. IL-17-producing cells may facilitate the development of CRC by assisting angiogenesis through the stimulation of VEGF production by cancer cells [194]. These cells have seen an increase in the intestinal mucosa of CRC patients due to microbial misbalance, which indicates that they can be a sensitive prognostic indicator for CRC [195]. In an experiment on serum and tissues of 99 samples and 37 controls, high IL-17 expression was seen, predicting IL-17 as a valuable tumor marker in CRC patients [196]. The variant of IL-17A can be utilized as a screening marker to assess CRC risk while its expression can be used as a biomarker for early CRC detection [197]. Moreover, an elevated level of Th17 cells was found in almost 80% of sporadic colon cancer tissues of humans, which indicates that IL-17 expression can be among potential biomarkers as prognostic entities for future developments in sporadic CRC [198].

### 5.3. Interleukin-22

Interleukin-22 has recently arisen as a novel part of CRC advancement as Th22 aggregation in patients showed relatedness with CRC advancement [199]. IL-22 in CRC tissue and serum or CRC tissue can be a prediction for the poor endurance of patients [14] elevating resistance to chemotherapy [200]. The polymorphisms in the IL-22 promoter are also linked with CRC risk [59]. Results of many studies suggest that IL-22 is involved in colon tumor maintenance as the analysis of IL-22 in human colon cancer showed that IL-22 mRNA expression in tumor tissue was more than two-fold higher than in normal tissue [201]. Their enhanced expression is related to the inflammation of colon mucosa in patients with gut infection or bowel diseases [202]. Excessive IL-22 in the cancer microenvironment leads to tumor growth with the activation of the STAT3 pathway [203], and the epigenetic activation of genes with a STAT3-dependent pathway maintains the CRC stem cells [59]. Levels of IL-22 in tumor tissues and blood are associated with chemoresistance and indicate a poor prognosis for patients having chemotherapy, so IL-22 may be a useful prognostic biomarker for CRC patients [200]. RORγt (necessary for IL-22 expression) and IL-17A expression (co-expressed with IL-22 sometimes) are associated with a bad prognosis of human CRC [171]. Modulation of IL-22 expression can also be due to various dietary components such as high fatty diet, and cruciferous vegetables; along with the microbiome, which has a substantial influence on IL-22 forming cells in CRC [14].

### 5.4. Interleukin-6

**Figure 4:**
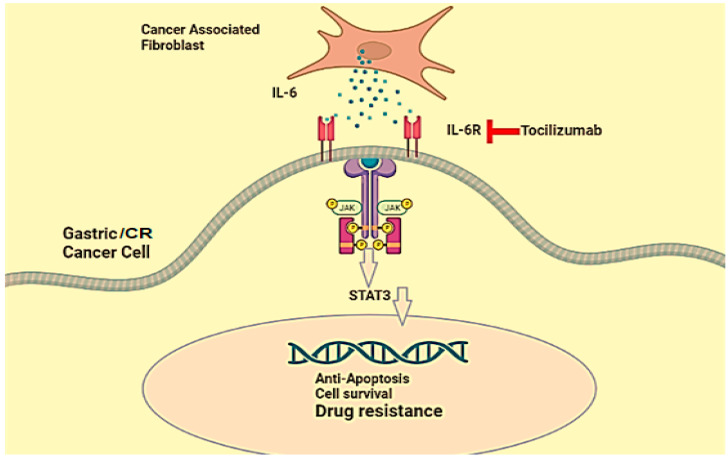
Schematic display of Tocilizumab that inhibits the activation of the Jak1-STAT3 signaling pathway in gastric/colorectal cancer cells. The Jak1-STAT3 pathway is activated by IL-6 (shown by colored dots in the figure).

Interleukin-6 (IL-6) is rapidly produced in response to tissue sprains and infections, contributing to host defense through immune reactions [201]. Studies on mice with colitis-associated cancer have found that treatment with anti-IL-6 receptor antibodies reduces the incidence of cancer, suggesting that IL-6 may be a therapeutic target for colorectal cancer (CRC) [202]. IL-6 plays a central role in the development of colonic cancer [58], with its expression significantly elevated in CRC tissues compared to non-cancerous cells and associated with an increased risk of relapse [202,203]. Several meta-analyses have indicated that serum IL-6 may be a potential biomarker for the diagnosis of CRC, and circulating IL-6 in plasma is also increased in patients with CRC [154,204,205]. Targeting the IL-6/STAT3 pathway has been proposed as a possible strategy for CRC therapy, as its expression can be an important factor in establishing prognostics for clinical decisions [206,207,208]. Recently, many therapeutic strategies have been developed that target the IL-6/STAT3 pathway for the treatment of CRC [209]. The IL-6/JAK/STAT3 signaling pathway drives the metastasis, proliferation, survival, and invasiveness of tumor cells in the tumor microenvironment by suppressing the antitumor immune response. Thus, targeting this pathway can directly inhibit cancer cell growth and stimulate the antitumor immunity [210]. Cancer-associated fibroblasts (CAFs) induce IL-6 to activate the Jak1-STAT3 pathway in gastric cancer cells by paracrine signaling. This allows tumor cells to progressively resist apoptosis, increasing their survival and resistance to chemotherapy. The humanized monoclonal anti-IL-6R antibody Tocilizumab (an FDA-approved drug) inhibits the activation of the Jak1-STAT3 signaling pathway, increasing the effectiveness of chemotherapeutic drugs [211]. Figure 4 illustrates its activity.

### 5.5. Interleukin-23

IL-23 is a heterodimeric type 1 cytokine composed of IL-12/p40 and p19 subunit, which is chiefly secreted by macrophages, monocytes, and activated dendritic cells and is vital in mucosal immunity [212,213]. Elevated levels of IL-23 have been found in colon adenocarcinoma, and it promotes tumor growth by blocking cytotoxic T cells and initiating pro-inflammatory responses [214]. IL-23 mRNA has been reported to be increased in various human tumors, correlating with a poor prognosis [214,215]. IL-23 influences tumor cells via T-cell responses by positively affecting the STAT3 activity in tumor growth, elevating TH17 activity and regulatory T cells (Tregs) [216]. Reports showed that IL-23 is highly expressed in tumor tissues of humans from other organs, and its expression is correlated with a poor prognosis [171,214]. IL-23R protein has been detected in the cancerous colorectal cell line SW-480, and its expression is progressively elevated from normal to colorectal cancer tissue [217,218,219,220,221]. The equilibrium between the cytokines IL-23 and IL-12 is a significant shift from inflammation to tumorigenesis [216,222].

### 5.6. Interleukin-33

IL-33 is a member of the IL-1 superfamily of cytokines expressed in various organ systems, including the gastrointestinal tract [222]. It was identified as a receptor ST2 ligand in 2005, and its mRNA processing forms multiple isoforms of proteins, including a secreted soluble form (sST2), a transmembrane receptor (ST2L), and a variant form [223]. Myofibroblasts, smooth muscle cells, fibroblasts, epithelial cells, adipocytes, and endothelial cells (non-hematopoietic cells) are the main sources of IL-33 production [224,225]. IL-33 is highly expressed in the serum of cancer patients and is also found in cancer cells and cancer-associated fibroblasts (CAFs) [226]. Many studies have reported its role in metastasis and tumorous cell invasion, and inhibiting it in colon cancer cells resulted in reduced tumor growth, migration in vitro, and fewer tumor cells in vivo [147,227]. Overexpression of IL-33 in cancer can increase the antitumor immune response by activating CD8+ T and natural killer cells [228]. Similar studies have depicted an increase in IL-33 in colorectal cancer compared to normal tissues, and its antitumorigenic effect in CRC [147]. There is a positive correlation reported between human CRC development and IL-33/IL-1RL1 expression levels [147,229], which reduces tumor growth in skin cancer and CRC models [230,231].

### 5.7. Interleukin-15

IL-15 is a cytokine that can activate CD8+ T cells and natural killer cells, leading to cytolytic activity [232]. This cytokine has shown the potential to enhance antitumor responses in cancer models [233,234]. The presence of IL-15 expression in the tumor microenvironment (TME) is crucial for optimal antitumor responses [235,236]. Its loss in expression is associated with low T cell proliferation, low T cell density, and decreased survival [235]. The mRNA expression of IL-15 has been detected in colorectal cancer cells of humans, such as Colo320, WiDr, TCO, and DLD1, through reverse transcriptase-polymerase chain reaction (RT-PCR) [237]. IL-15 may have the potential to be used in cancer therapy, as it exhibits strong immune stimulatory functions in addition to its role as a growth factor that regulates homeostasis and lymphocyte function [238]. IL-15 has antitumor effects by activating the cytotoxicity of natural killer cells and producing other cytokines such as TNF-α and IFN-γ [239]. Deletion of IL-15 from CRC tissues results in fewer T cells compared to tumors where IL-15 is not deleted, as IL-15 induces T cell proliferation. Therefore, IL-15 deletion can be utilized as a prognostic biomarker in CRC [240].

### 5.8. Interleukin-18

The expression of IL-18 is reported to be low in colon cancer tissues and may be associated with tumor size, while also suppressing the proliferation of colon cancer [133]. Known as the “IFN-γ-inducing factor,” IL-18 induces IFN-γ expression in mice when treated with lipopolysaccharide [241]. The protein encoded by the IL-18 gene, located at 11q23.1, is responsible for pathogenic response and activation of host defense mechanisms [242]. IL-18 is mainly secreted by dendritic cells and macrophages, stimulating the production of interferon-γ (IFN-γ) by thymus-dependent lymphocytes (T cells) and natural killer cells (NK) [243]. IL-18 expression is elevated in the blood serum of CRC patients and is associated with tumor size, histological grade, and cancer cell metastasis, making it a potential indicator to predict CRC patients’ prognosis and survival time [143]. Gene expression of IL-8 is elevated (*p* < 0.05) in CRC patients compared to healthy individuals, detected using ELISA and real-time PCR [143].

### 5.9. Interleukin-13

IL-13 and IL-4 receptors may become attractive targets for the treatment of colorectal cancer [244,245]. High levels of IL-13Rα2 were detected by immunoblotting in metastatic colon cancer cell lines, and 66% of tumor samples in patients showed clear overexpression of IL-13Rα2 [246]. High levels of IL-13 and IL-13R expression are seen in 50% of Stage I–III CRC patients and are associated with longer survival time [244]. Its appearance is also related to high tumor stage and poor human CRC outcomes [246]. A study showed that IL-13 serum levels were significantly lower in advanced-stage patients, which are associated with a poorer prognosis [247]. Yet, another study with fecal samples showed higher IL-13 levels in 20 CRC patients compared to 20 healthy individuals [248]. IL-13 enhances the expression of EMT-promoting factor ZEB1 with a positive correlation between IL-13Rα1 and ZEB1 at mRNA levels in human CRC samples. Hence, the IL-13/IL-13Rα1/STAT6/ZEB1 pathway plays a critical role in promoting EMT and CRC aggressiveness [249]. Reports showed that IL-13R is involved in the local metastases process of colorectal cancer, while expression of IL-13 has an impact on survival. These interleukins and their receptors may become attractive targets for the treatment of colorectal cancer. [244]. Both expression level of IL-13Rα2 and IL-13Rα2-mediated signaling has been reported to cause cell survival, tumor proliferation, tumor progression, invasion, and metastasis [250].

### 5.10. Interleukin-4

Interleukin-4 (IL-4) is an anti-inflammatory and immunomodulatory cytokine that promotes cancer-directed immune surveillance [244]. IL-4R is expressed in human gastric cancer cell lines, such as CRL1739, and its expression contributes to local metastasis in colorectal cancer, making it an attractive target for CRC therapy [244]. IL-4 and IL-13 are cytokines that are structurally and functionally related, sharing common receptor subunits. They regulate immune responses and play a role in various human cancer pathogenesis, chemosensitivity, and prognosis [245,246,247]. The T allele of IL-4 rs2070874 is associated with a higher risk of gastrointestinal cancer [248]. IL-14 activates tumor-associated macrophages and suppressor cells containing tumor-promoting functions [251]. IL-4 is involved in the promotion of epithelial-to-mesenchymal transition (EMT) in CRC [146], while it inhibits the growth of GC cells, and its growth inhibitory effects are positively related to IL-4R expression in cell lines [252]. Moreover, IL-4 promotes EMT in CRC cell lines “HCT 116” and “RKO” through the STAT6 pathway [146].

### 5.11. Interleukin-8

Interleukin-8 (IL-8) is a chemokine that belongs to the CXC cytokine family and is markedly upregulated in colorectal cancer (CRC), contributing to enhanced invasion, tumor growth, and metastasis [253,254]. IL-8 has diverse biological actions, including promoting inflammation, infectious diseases, invasion, migration, angiogenesis, and proliferation [255]. Serum IL-8 levels are a promising biomarker for detecting CRC and identifying high-risk patients [256]. IL-8 induces CRC cell migration and proliferation through the ADAM-dependent pathway and disintegrin, where heparin-binding epidermal growth factor (EGF) acts as a major ligand [257]. IL-8 has a multifunctional role in CRC progression, including enhancing the survival of cancer cells, promoting tumor cell proliferation, and regulating adhesion and invasion [258,259,260]. Excessive expression of IL-8 in the cancer microenvironment promotes colon cancer growth and metastasis, but the absence of its receptor CXCR2 prevents cell growth [261]. The autocrine properties of IL-8/CXCR2-mediated activation facilitate the intrinsic mechanism of tumor cells to avoid stress-induced apoptosis [262]. Increased plasma levels of IL-8 are associated with a single-nucleotide polymorphism (SNP) in IL-8 at 251 bp upstream [263], with the IL-8T–251A polymorphism being individually associated with tumor reappearance risk [264]. IL-8 also regulates chemosensitivity and angiogenesis (in vitro and in vivo) in models of colon cancer [265]. Stage IV CRC shows almost 10-fold higher serum levels of IL-8 than normal individuals [265]. Enhanced serum expression of IL-8 is also found in liver and lung tissue damage along with metastatic CRC [265], suggesting that the systemic increase in IL-8 can be more important for prognosis than the local and cancer cell-derived IL-8 levels. IL-8 is highly expressed in CRC tissues but is differentially produced by tumor components depending on the genetic background of CRC. As IL-8 is a strong prognostic factor in CRC, it may be used for prognostic assessment and tailoring of therapeutic strategies in individual CRC patients [266].

### 5.12. Interleukin-11

Interleukin-11 (IL-11) belongs to the IL-6 family and has a wide range of functions, including hematopoiesis, bone development, tissue repair, and tumor development [267,268]. In some cancer cells, the IL-11 receptor (IL-11R) has been identified, which contains IL-11Rα1. When bound to IL-11 and gp130, this receptor transmits signals to the nucleus through Janus kinase (JAK) activation [269,270]. JAKs subsequently phosphorylate STAT3, which then enters the nucleus and activates the transcription of numerous target genes involved in the suppression of cell proliferation and apoptosis [158,271]. IL-11 production is regulated by various cytokines, such as TGFβ, IL-1β, IL-17A, and IL-22 [272,273,274,275]. A human study has suggested that a polymorphism of the IL-11 gene is linked to an increased susceptibility to ulcerative colitis (UC) in patients [276]. While mild UC patient’s exhibit increased IL-11 expression, severe UC patients show a decrease in expression [277]. A study using a human cancer database found that genes enriched in IL-11+ fibroblasts were elevated in human colorectal cancer, and the high expressions of several of these genes correlated with a reduced disease-free survival rate in colorectal cancer patients [278]. Previous studies have demonstrated that IL-11+ cells are derived from stromal or epithelial cells [53,158]. There is substantial evidence that IL-11 regulates tumor progression, cellular growth, and differentiation. While IL-11 has been suggested to become a therapeutically important molecule in the supportive care of cancer patients receiving chemotherapy [278], the study indicates that IL-11 may upregulate colorectal carcinoma cell growth and/or invasion, necessitating cautious attention to the therapeutic use of IL-11 [279]. Table 3 contains detailed description of interleukin families involved in CRC and their potential therapeutic strategies.

## 6. Discussion

Despite significant advances in treatment, mortality from colorectal cancer remains high, and 40–50% of patients eventually die due to the disease. The most significant impact on its incidence and mortality will come from extensive population screening [334]. Colorectal cancer is a complex and diverse group of disorders at the molecular level, involving signaling pathways with different patterns of genetic mutations [335]. Epigenetic modifications cause the progression of the disease along with the responses to specific therapies. As it is caused by the activation of multiple signaling pathways and cannot be targeted with a single treatment, combinations of conventional therapies with advanced inhibitors are immediately needed to target dysregulated pathways. It has been extensively recognized that immune system dysfunction, including abnormally expressed cytokines, is strongly associated with the progression and pathogenesis of colorectal cancer [336]. Diagnostic tests have many limitations. For example, fecal blood test screening suffers from low sensitivity for polyps, and colonoscopy is invasive [337,338].

The most important feature of activated immune cells is the production and release of growth factors and cytokines that modulate the inflammatory conditions [339,340,341,342,343,344,345,346]. Cancer-associated inflammation is a determining factor in disease progression and survival in CRC, contributing to invasion, tumor angiogenesis, and metastatic spread [347,348]. CRC biomarkers can be divided into two groups based on clinical criteria: “diagnostic biomarkers” for the detection or confirmation of the presence of the disease, and “clinical biomarkers” for the prediction of patients’ response to a specific treatment or their prognosis [349]. Although many novel therapeutic improvements have been anticipated to treat colon cancer, the survival rate is still unsatisfactory due to metastasis and tumor reappearance [44]. Molecular biomarkers are being explored for implementation in clinical practice in this period of precision cancer medicine [350]. These prognostic biomarkers are desired to assist patients and predict survival. A deeper understanding of CRC is required, and the efficiency of targeted therapies and the development of more efficient biomarkers provide an encouraging prospect for the future management of CRC [349]. We believe that with the discovery of more novel targeted therapeutics, the disease burden of CRC can be decreased in the future [351]. However, there have been only a few studies on point-of-care cytokine detection due to challenges such as low concentration, complex secretion process, thermal instability, and others [29,74]. Yet, studies have investigated the use of ILs as biomarkers for monitoring the response to treatment in CRC patients [12,352,353]. These studies demonstrate the potential of ILs as biomarkers for the diagnosis of CRC and these findings are based on studies that have been conducted on a small number of patients and more research is needed to confirm the accuracy of these results in larger patient populations.

Finding more effective prognostic markers and therapeutic targets for patients with advanced colorectal cancer [354] is important because the majority of patients with advanced colon cancer cannot undergo surgery. Subsequently, due to the widespread adoption of CRC screening in the population, many patients would be diagnosed at the preclinical stage through screening [355]. Given the rise in treatment costs, screening for colorectal cancer is a cost-saving tool in many countries [356]. Various genomic projects have acknowledged new potential molecular markers and targets for colorectal cancer to guide more specific treatments for patients [357]. Oncogenes involved in CRC are mainly well characterized; nevertheless, the effects of additional environmental factors in this disease are undefined. Molecular biomarkers have been investigated for the last 20 years with promising results. However, many drawbacks affect the consistency of the conclusions [358,359,360,361]. More detailed research is needed on the relationship between diet, microbiota, and CRC.

There is high heterogeneity and complexity in CRC; therefore, standard treatments including radiation/chemotherapy are only effective in only a few patient populations. Tumors can also have various core genetic causes which makes the protein expressions different in each patient along with their responses to generic treatments [362]. This intrinsic changeability of cancer lends to the growing field of precision and personalized medicine (PPM). Many steps are being taken in order to attain PPM data to distinguish molecular differences between tumors. These include “immunotherapy” to utilize the patient’s own immunity against cancer, containing cytokines, vaccines, checkpoint inhibitors, monoclonal antibodies (mAbs), and hematopoietic stem cell transplants (HSCTs) [363]. There is a growing category of PPM products known as “companion diagnostics (CDx)”, molecular assays that assess proteins, genes, or specific mutation levels to diagnose and suggest a specific and effective therapy for an individual’s condition [364].

## 7. Future Perspective

Extensive population screening is expected to have the most significant impact on colorectal cancer incidence and mortality. By developing and implementing new, more specific and sensitive biomarkers, clinicians can improve diagnostic strategies and detect CRC cases early in the disease, thereby improving the prognosis of thousands of patients. Several new potential molecular targets and markers for colorectal cancer have been identified through various genomic projects, providing guidance for more specific treatments. Although most of the major oncogenes involved in CRC are well characterized, the effects of additional environmental factors in this disease are still undefined. While molecular biomarker studies over the past two decades have shown promising results, some drawbacks limit the reliability of the conclusions. Therefore, further research is needed to investigate the relationship between diet, microbiota, and CRC in greater detail.

## 8. Conclusions

Previously, scientists have reported significant information on the various genes and proteins that contribute to cancer. Mutated genes and the identification of related environmental factors are key discoveries. Using molecular methods, important gene expressions can be determined and used as novel biomarkers to reduce cancer complications and treatment. Further studies are required to explore the pathways and mechanisms involved in the expressions of immunological biomarkers and their involvement in the development and progression of colorectal cancer. Detecting cytokines at the required detection limit for reliable results is challenging, but many efforts have been made to develop cytokine assays with more sensitivity. Research into cytokine quantification is still developing to find effective solutions for the accurate and real-time detection of multiple cytokines in vivo. The effects of promising targets on different immune cell populations are still poorly understood. Therefore, improving antitumor responses and suppressing immune cells that support tumor growth are the prospects for cytokine-based cancer treatment. However, identifying all environmental factors, pivotal genes, immune responses, and cytokine release at the cancer stage provides a comprehensive map for further efforts to reduce cancer in the future.

## Figures and Tables

**Table 1:** Cancer cell types in TME and the specific soluble factors released by them. Soluble factors involved in the promotion of cancer stem ness pathways.

TME Cells	Soluble Factors	Target Cells	Signaling Pathway	Biological Effects	Reference
CD4+	IL-22	CRC	STAT3/DOT1L (Signal transducer and activator of transcription)/(Disruptor of telomeric silencing 1-like)	Stemness gene regulation	[59]
CAF (Cancer-associated fibroblasts)	HGF/SDF1 (Hepatocyte growth factor)/Stromal cell-derived factor-1	Cancer stem cells (CSC)	Wnt/β-catenin	Clonogenic activity and expression of CD44v6	[60]
Endothelial cells	JAG1 (Jagged)	CRC	Notch	CD133 expression, tumorigenicity and chemoresistance	[61]
MSC (Mesenchymal stem cells)	PGE2 (Prostaglandin E2)	CRC	Wnt/β-catenin	EMT (Epithelial-to-mesenchymal transition) and invasion	[62]
Myofibroblasts	HGF	CSC	Wnt/β-catenin	Clonogenicity	[63]

**Table 2:** Major interleukins involved in CRC progression and studied as biomarkers.

Cytokine	Functional Effect in CRC	Expression Patterns	Reference
IL-1α	Promotes metastasis and the chemosensitivity	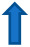	[136,137]
IL-1β	Promotes the proliferation of colon cancer cells, tumorigenesis, and alters the tumor microenvironment	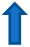	[137,138,139]
IL-18	Antitumorigenic properties and release of other signals	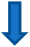	[140,141]
IL-2, IL-7, IL-9, IL-15	Antitumor activity, promote EMT, proliferation, invasion, and metastasis	IL-4, IL-7 upregulated, IL-9 downregulated, IL-2 in between	[142,143,144,145,146,147]
IL-21	Activation of immune response biomarkers	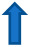 (Potential for biomarker)	[148,149,150]
IL-6	Promotes mitosis, proliferation, metastasis, migration, and angiogenesis	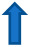	[151,152,153,154]
IL-11	Facilitates the proliferation of CRC	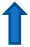	[155,156,157]
IL-8	Promotes cell proliferation, angiogenesis, cancer metastasis, chemoresistance, antianoikis, maintains CCSC properties	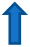	[158,159,160]
IL-10	Pathogenesis and progression	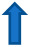	[141,161,162,163]
IL-22	Dominant role in CRC tumorigenesis, antiapoptosis, and cell proliferation	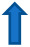	[164,165,166]
IL-17a	Promotes cell cycle progression and angiogenesis	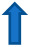	[167]
IL-17b	Promotes tumor	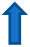	[168,169,170,171]
IL-4	Overexpressed in early CRC, tumor development	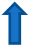	[172]
IL-23	Overexpressed in CRC tissue and predictive for CRC metastasis	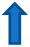	[173,174,175,176]

Upwards arrow just showed upregulation of the genes while downwards show suppression or downregulation.

**Table 3:** Interleukin families in colorectal cancer.

Interleukin Family	Receptors	Cytokine	Potential Effect in CRC	Therapeutic Strategy	Reference
	IL-1R1 IL-1R2	IL-1α	Metastasis promotion with chemosensitivity. Promotes antitumor immunity and carcinogenesis (inflammatory)	Therapeutic neutralization in order to tackle severe illness in clinical trials	[102,139,140,280]
	IL-2R1 IL-1R2 ILR3 sIL-IR2 sIL-IR3	IL-1β	Proliferation of cancer cells of colon and promotion of tumorigenesis. Altering the tumor microenvironment	Therapeutic neutralization to manage cytokine release syndrome (CRS) in clotting time and the prevention of cancer	[102,140,141]
IL-1 super family	IL-1R4 (ST2)	IL-33	Protumor, maintenance of intestinal microbiota, tumor microenvironment change, TH2 polarization, Treg cell function, promotion of Angiogenesis and enhancement of colon cancer cell stemness, maintain intestinal microbiota.	Preclinical neutralization	[15,281,282,283,284]
IL-18 subfamily	IL-1R5–IL-1R7 IL-18BP IL-1R5 (IL-18Rα IL-18Rβ)	IL-18	Antitumor, activates lymphocytes to produce IFN-γ, improve the integrity of intestinal barrier and induces apoptosis to act on NK cells	Preclinical engineered rIL-18 or by combined with activated clotting time, hindered by IL-18BP	[140,285]
	IL-1R8–IL-1R5	IL-37	Antitumor attributes, inhibit the colon cancer cell development by stopping β-catenin.	Not explored	[135,286]
	IL-1R6	IL-36α	Antitumor		[287]
IL-36 subfamily	IL-1R6	IL-36γ	Antitumor, inflammatory immune infiltrates promotion, promote inflammation (TH1-type) inhibited by IL-36Ra	Preclinical rIL-36γ as an alternative to IL-1	[287]
	IL-38	IL-1R6–IL-1R9	Immunosuppressive	Not explored	[288,289]
	IL-2Rα, IL-2Rβ/IL-2Rγ, IL-2Rα/IL-2Rβ/IL-2Rγ sIL-2RαIL-2/IL-15Rβ–γc IL-2Rα–IL-2/IL-15Rβ–γc	IL-2	Antitumor, NK and T cell growth factor, inhibit T cell responses by maintaining Treg cells and AICD induction	rIL-2 approved for monotherapy. Engineered to avoid side effects and to be used in ACT	[147,148,290]
	Type (IL-4Rα/γc) and Type (IL-4Rα/IL-13Rα1)	IL-4	Promote epithelial-to-mesenchymal transition (EMT), metastasis and invasion. Promotes inflammation of TH2-type and polarization of TH9. Promotes cancer cell growth upon overexpression of IL-4R.	IL-4R- targeting to bear cancer cells and block signaling. Antitumor TH9 cells production for ACT	[146,291]
IL-2 family	IL-7R (IL-7Rα/γc)	IL-7	Metastasis promotion Antitumoural: NK growth factor and T cell production	rIL-7 in combination with interleukins or ACT	[145,292,293]
	IL-9R (IL-9Rα/γc)	IL-9	Antitumor action Pleiotropic	Preclinical TH9 cells in ACT	[15,109,294]
	IL-15R (IL-15Rα/IL-15Rβ/γc)	IL-15	Proliferation and angiogenesis inhibition, Antitumor activity by activating lymphocytes to produce IFN γ, Promote apoptosis	rIL-15 or analogues in combination with interleukins or ACT	[295,296]
	IL-21R–γc heterodimers of IL-21R and γc	IL-21	Enhances cytotoxicity of CTLs, Antitumor activity	Combination therapies with rIL-21 in clinical trials	[296]
IL-3 family	IL-3Rα–βc	IL-3	Promotes malignancy as a haematopoietic factor	Target CD123-bearing cells by fused to toxins	[297,298,299]
IL-6 family	gp130 IL-6R IL-6Rα–gp130 (classic) sIL-6Rα–gp130 (trans)	IL-6	Mitosis promotion, metastasis, proliferation, making the microenvironment for metastasis, activates tumor outgrowth and carcinogenesis, mediates cytokine release syndrome (CRS), Cachexia promotion	Neutralization to manage CRS in ACT, cachexia	[300,301,302,303]
	gp130 IL-11Ra IL-11Rα–gp130 (classic) sIL-11Rα–gp130 (trans)	IL-11	Proliferation of CRC Promotes inflammation by inducing carcinogenesis and cancer progression	Preclinical neutralization and gp130 common receptor blockade	[158,304]
	IL-31Rα–OSMRβ	IL-31	TH2-type cytokine, evidently tumorigenic	Unexplored	[287]
IL-10 family	IL-10RA and IL-10RB	IL-10	Promotes cytotoxicity, inhibits antitumor responses	rIL-10 to increase cytotoxicity in trials	[163,165,305,306]
	IL-10RB and IL-22R /IL-22BP	IL-22	Promote tumorigenesis, antiapoptosis and cell proliferation Peritumoral: promotion of carcinoma progression	Preclinical neutralization	[114,306,307,308]
	IL-20Rα–IL-20RβIL-22Rα1–IL-20Rβ	IL-24	Induces autophagy of cancer and apoptosis	Preclinical rIL-24 combined with oncolytic virus	[309,310]
	IL-20Rα–IL-10Rβ	IL-26	Pro-tumoral through TH17 cells and neutrophils	Preclinical neutralization	[311,312]
	IL-12Rβ1–IL-12Rβ2	IL-12	Antitumoral: the main driver of TH1-type immunity, amplification and initiation of production	Engineered or combined with other interleukins in trials	[286,313]
IL-12 family	IL-23R–IL-12Rβ1	IL-23	Mainly pro-tumoral: direct and indirect effect via TH17 cells and TH22 cells	Neutralization in trials, enhances CAR T cell cytotoxicity	[314,315]
	IL-27Rα	IL-27 and IL-30	Pleiotropic: induces cytotoxicity and NK cell yet enhances T cell and Treg cell activity	Neutralization and engineered rIL-27 in trials	[316,317,318,319]
	IL-12Rβ2–gp130gp130–gp130IL-27Rα–IL-12Rβ2	IL-35	Treg cell-mediated suppression of T cell responses and promotion of metastatic colonization	Preclinical neutralization with checkpoint inhibitors and other therapies	[320,321]
IL-17 family	IL-17RA–IL-17RC	IL-17A/F	Cell cycle progression and angiogenesis, facilitate the development indirectly and change the tissue environment and microbiota of CRC	Neutralization in clinical trials	[169,322]
	IL-17RIL-17RB	IL-17b	Carcinogenesis, immunosuppression, EMT		[170,323]
	IL-17RA–IL-17RE	IL-17c	Mostly pro-tumoral, but antitumoral properties	Not explored	[324]
	Unknown	IL-17 D			[325]
	IL-17R	IL-17e			[170]
	IL-17RA–IL-17RB	IL-25			
	IL-17R	IL-17f	Tumor suppression effect possibly by inhibiting tumor angiogenesis		[326]
IFN-γ family	IL-28A and IL-28B IL-28Rα–IL-10Rβ	IL-28Rα IL-10Rβ IL-29	Antitumoral: induces apoptosis of malignant cells	Preclinical gene therapy using IL-28 and IL-29	[327]
	CXCR1, CXCR2ACKR1/DARC	IL-8	Attracts neutrophils and mediates the suppressive environment	Therapeutic neutralization in clinical trials	[328]
	IL-13Rα1–IL-4RαIL-13Rα2		TH2-type cytokine	Targeting or blocking IL-13R	[291]
	IL-14α and IL-14β	IL-14R	Growth factor in B cell and in lymphoma	Not explored	[329]
	CD4	IL-16	Pro-tumoral: proliferation of lymphoma and chemoattractant	Scarce preclinical evidence	[330]
Other interleukins	Unknown	IL-32	Pleiotropic in action but depends on cancer type and isoform.	Preclinical antitumor effects in combination	[331]
	CSF1R	IL-34	Pro-tumoral: immune suppression, cancer progression, and resistance of therapy	Preclinical neutralization to lessen the pro-tumor effects	[332,333]

## Data Availability

Not applicable.
